# Obstructive-Type Jaundice without Bile Duct Dilatation in Generalized Peritonitis Is a Specific Sign of Spontaneous Gall Bladder Perforation

**DOI:** 10.1155/2020/6504260

**Published:** 2020-09-22

**Authors:** Vicky Sumarki Budipramana, Maria Meilita

**Affiliations:** ^1^Division of Digestive Surgery—Department of Surgery, Dr. Soetomo Genetral Hospital, Universitas Airlangga, Surabaya, Indonesia; ^2^Department of Surgery, Dr. Soetomo General Hospital, Universitas Airlangga, Surabaya, Indonesia

## Abstract

Generalized peritonitis caused by spontaneous gall bladder perforation is rare and difficult to diagnose preoperatively. The increased absorption of the spilling conjugated bile from the gall bladder by the inflamed peritoneum causes jaundice and direct hyperbilirubinemia. A 56-year-old male patient with septic generalized peritonitis and obstructive-type hyperbilirubinemia came to our hospital. The blood examination showed that total, direct, and indirect bilirubin were 6.20, 5.38, and 0.82 mg/dl. The gall bladder perforation was not detected on USG, CT scan, and MRCP. On laparotomy, we found a 0.5 cm perforation on the fundus of the gall bladder and 2500 cc of bilious fluid in the peritoneal cavity. Cholecystectomy was performed, and the patient completely recovered after the surgery. Preoperative diagnosis of spontaneous gall bladder perforation is difficult even by using ultrasonography, CT scan, and MRCP. The inflammatory reaction of the peritoneum in bile peritonitis increases the absorption of the spilled conjugated bile from the peritoneal cavity. The obstructive-type hyperbilirubinemia without dilatation of the bile duct on imaging examination was the sign of bile leakage into the peritoneal cavity. In septic condition, the preoperative diagnosis of the spontaneous gall bladder perforation is important to establish so that the surgeon can make only a minimal procedure in reducing the morbidity. Therefore, it could be concluded that the presently occurring obstructive-type jaundice without dilatation of the bile duct in the case of generalized peritonitis is a specific sign of spontaneous gall bladder perforation.

## 1. Introduction

Preoperative diagnosis of spontaneous gall bladder perforation is difficult to make even with imaging examination, such as USG, CT scan, and or MRI [1–3]. The dilution of the previously hyperosmotic and irritative alkaline bile by the peritoneal fluid and the increased absorption of the conjugated type of bilirubin in the inflamed peritoneum cause direct hyperbilirubinemia [4, 5]. The combination symptoms of peritonitis and obstructive-type jaundice at the same time and no dilatation of the bile duct on imaging examination are the specific signs in making the diagnosis of spontaneous gall bladder perforation. In septic condition, the preoperative diagnosis of spontaneous gall bladder perforation is important to make so that the surgeon can decide a more minimal procedure to reduce the morbidity.

## 2. Case Report

A 56-year-old man came to the emergency room suffering from pain in the whole abdominal region, fever, and jaundice since two days before. The vital signs were the BP 90/60 mmHg, HR 110, and rectal temperature 38.6°C. The patient had jaundice, and the abdomen was distended, guarding with rebound tenderness and decreased bowel sound. The laboratory values were as follows: white blood cell count 25.020 mm^3^; total bilirubin 6.20 mg/dl; direct bilirubin 5.38 mg/dl; indirect bilirubin 0.82 mg/dl; AST 22 U/L; ALT 15 U/L; amylase 18 U/L; and lipase 13,28 U/L.

**Figure 1:**
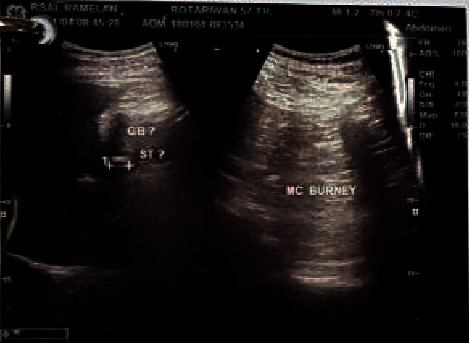
Abdominal ultrasound: normal gall bladder with stones without dilatation of the bile duct.

**Figure 2:**
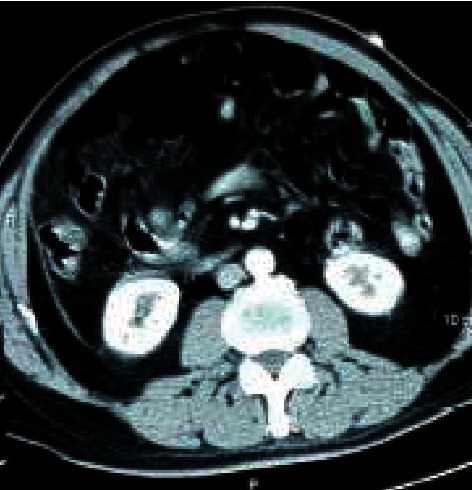
Abdominal CT scan: nonconclusive finding.

On abdominal ultrasound, the gallbladder was normal with stones inside, with the biggest size 1.29 cm without dilatation of the bile duct system (Figure 1). There was also nonconclusive finding on abdominal computed tomography (Figure 2).

**Figure 3:**
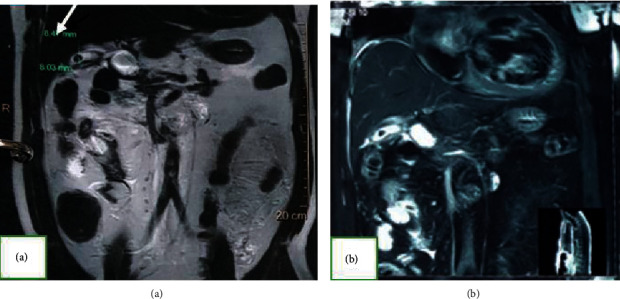
Abdominal MRI: (a) gall bladder with the stone inside (arrow) and free peritoneal fluid; (b) no dilatation of the intrahepatic bile duct (IHBD) and extra hepatic bile duct (EHBD).

MRI was performed due to the jaundice and direct hyperbilirubinemia, and the result showed that there were two gallstones, without dilatation of IHBD and EHBD (Figure 3). The preoperative diagnosis was septic generalized peritonitis with suspected gall bladder perforation.

**Figure 4:**
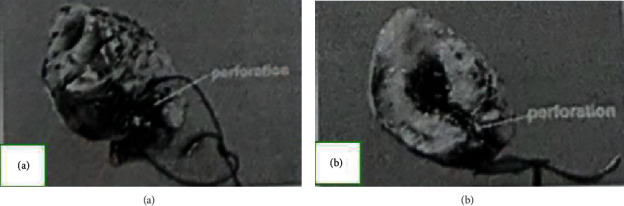
Gall bladder specimen: (a) perforated tract marked with a thread; (b) gall bladder section through the tract.

On laparotomy, we found a 0.5 cm perforation on the fundus of the gall bladder and 2500 cc of bilious fluid in the peritoneal cavity. Laparotomy and cholecystectomy were performed. On the specimen examination, we found a perforated tract on the fundus of the gallbladder surrounded by the focal necrotic area (Figure 4). After being operated on, the patient completely recovered and allowed home 7 days after the surgery.

## 3. Discussion

The etiopathogenesis of spontaneous gall bladder perforation is still obscure; however, the most plausible mechanism is the complication from the stones and acute cholecystitis [6, 7]. The clinical sign presented was like acute cholangitis because the patient was having jaundice, fever, and abdominal pain with the total bilirubin 6.20 mg/dl and direct bilirubin 5.38 mg/dl. The sign of infection WBC 25.02 k/uL and CRP 235.00 mg/l was also found. However, after we found that there was no dilatation of the bile duct on MRCP, the diagnosis of acute cholangitis was excluded. Generally, the signs and symptoms of spontaneous perforation of the gallbladder show that it is difficult to distinguish them from peritonitis because of other causes [8–11].

Ultrasonography was not conclusive in the presumptive diagnosis of gall bladder perforation even with CT scan and MRI. The HIDA scan is more sensitive to diagnose gall bladder perforation; however, it is not suitable for patients with septic peritonitis and unstable hemodynamics [12].

The inflammatory reaction of the peritoneum in bile peritonitis induces neoangiogenesis which increases the effective endothelial surface layer leading to relatively faster absorption of the spilled conjugated bile from the peritoneal cavity to the blood capillaries [4]. Moreover, the dilution of the previously hyperosmotic and irritative bile by the peritoneal fluid makes the osmotic conductance of the bile through the peritoneal membrane increase [5]. Bile consists of both conjugated (direct) and unconjugated (indirect) bilirubin in varying proportions [13]. The increased absorption of the conjugated bile in the peritoneal cavity by the inflamed peritoneum in the case of bile peritonitis causes the patient to have jaundice and direct hyperbilirubinemia on blood examination.

In the case of bile peritonitis, indirect bilirubin is less absorbed by the peritoneum because it predominantly exists in its diacid form rather than to the smaller amounts of mono/dianion. The diacid form has low aqueous solubility and tends to aggregate on the lipid membrane [14]. On the contrary, the direct bilirubin is water-soluble [15]. Therefore, the direct bilirubin increased its systemic concentration greater than indirect bilirubin, mimicking obstructive-type jaundice in this patient.

The acidity (pKa) also affects the bioavailability of bilirubin. The direct bilirubin pKa is approximately 1.5, whereas the indirect bilirubin pKa varies widely from 4 to 9 [16]. At the pH of the peritoneal fluid, direct bilirubin will be ionized and more water-soluble than indirect bilirubin is, and therefore, the former is potentially absorbable [17].

Spontaneous common bile duct perforation also shows the similar sign, that is, obstructive-type jaundice. However, jaundice should have begun long before the sign of generalized peritonitis because it must be preceded by the previous common bile duct obstruction [18]. In this case, jaundice presently occurred concomitant with the signs of generalized peritonitis, so we excluded the common bile duct perforation. Hyperbilirubinemia is also found in generalized peritonitis caused by perforated or gangrenous appendicitis; however, in this case, not only the direct hyperbilirubinemia but also concomitant with indirect hyperbilrubinemia may occur. The pathogenesis is thought to be because of bacteremia or endotoxemia brought through the portal vein causing an imbalance between the production and excretion of bilirubin in the liver, and it impaired the excretion of bilirubin from the bile canaliculi [19, 20]. There are also the cases with other peritonitis caused by bowel perforations where the infection is also brought through the portal vein to the liver and causes both direct and indirect hyperbilirubinemia [21]. However, in the case of spontaneous gall bladder perforation, only direct hyperbilirubinemia may occur, but indirect hyperbilirubinemia does not increase much because the infection of the gall bladder that causes perforation to be brought not through the portal vein. Moreover, indirect bilirubin is less absorbed by the peritoneal membrane.

It should be emphasized that the symptom of peritonitis concomitant with obstructive-type jaundice and no dilatation of the bile duct are the specific signs in making the diagnosis of gall bladder perforation. However, when there is a dilatation of the bile duct, then the diagnosis of acute cholangitis is suspected.

The preoperative diagnosis of spontaneous gall bladder perforation in the case of septic peritonitis is important to make so that the surgeon can decide not to perform more invasive surgery. In septic condition, only performing percutaneous biliary drainage is more beneficial than immediately performing laparotomy and cholecystectomy. By draining the bile from the peritoneal cavity, the condition of the patient will improve. Then, on the next step, cholecystectomy can be performed with less morbidity without performing laparotomy [22].

From the abovementioned explanation, it could be concluded that the presently occurring jaundice and obstructive-type hyperbilirubinemia without dilatation of the bile duct in the case of generalized peritonitis are specific signs of spontaneous gall bladder perforation. In septic condition, the preoperative diagnosis of spontaneous gall bladder perforation is important to establish so that the surgeon can make only a minimal procedure in reducing the morbidity.
